# Prediction of Corrosion-Induced Longitudinal Cracking Time of Concrete Cover Surface of Reinforced Concrete Structures under Load

**DOI:** 10.3390/ma15207395

**Published:** 2022-10-21

**Authors:** Jian Wang, Yongyu Yuan, Qiang Xu, Hongtu Qin

**Affiliations:** 1 School of Civil Engineering, Sun Yat-sen University, Guangzhou 510275, China; 2 Research Institute of Highway Ministry of Transport, Beijing 100088, China

**Keywords:** steel corrosion, rust-filling layer, thick-walled cylinder model, critical corrosion depth, prediction model

## Abstract

Reinforced concrete (RC) structures suffer from different types of loads during service life, and the corrosion characteristics of steel bars embedded in concrete under load are different from those under non-load. In this paper, when the interface between steel bars and concrete (IBSC) cracked and the concrete cover surface (CCS) cracked, the effects of load on the critical corrosion depth of steel bars were analysed based on the thick-walled cylinder model, and a prediction model for the corrosion-induced longitudinal cracking (CLC) time (i.e., initiation cracking time) of the CCS of RC structures under load was proposed. Finally, the influence of load on the CLC time of CCS was discussed on the basis of the proposed prediction model. The results showed that the load had a significant effect on the critical corrosion depth of steel bars when the IBSC cracked induced by corrosion, while the influence of load on the critical corrosion depth of steel bars when the CCS cracked induced by corrosion was not obvious. When the CCS cracks induced by corrosion under load, the influence of the rust-filling layer on the critical corrosion depth of steel bars was larger than that of the load. With the increase in load, the CLC time of CCS decreased. The calculated values of the proposed prediction model were in reasonable agreement with the experimental values, which can provide a reference for durability evaluation and service life prediction of RC structures and lay the foundation for the investigation of the corrosion depth of steel bars in concrete under load.

## 1. Introduction

In coastal, marine, or other chloride environments, chloride ion erosion is the main cause of steel corrosion in reinforced concrete (RC) structures, and corrosion further leads to the degradation of the durability of RC structures [1,2]. The chloride ions in the environment reach the surface of steel bars through the pores and micro-cracks inside the concrete, and their concentration is continuously accumulated on the reinforcement surface. When the chloride ion concentration reaches a threshold, the dense passive film on the surface of the steel bars is destroyed, and then, with the presence of oxygen, the local corrosion is activated. Subsequently, the steel bars undergo electrochemical corrosion, that is, the steel dissolution reaction of the anode and the oxygen consumption reaction of the cathode occur simultaneously on the surface of the steel bars [3]. Generally, chloride ion erosion will cause non-uniform corrosion of steel bars, which is serious on the side of steel bars facing the concrete cover surface (CCS). Non-uniform corrosion can significantly reduce the strength and ductility of steel bars [4,5] and then lead to the degradation of the load-bearing capacity, stiffness, and ductility of RC structures [6]. There are many factors affecting the corrosion of steel bars in concrete, such as the water-binder ratio or concrete strength, mineral admixture, cement type, concrete cover thickness, reinforcement type and diameter, temperature and relative humidity, chloride ion concentration, external load, crack width, etc., [7,8,9,10,11,12] and it should be noted that under different conditions, the main influencing factors of steel corrosion will be different.

After the corrosion of steel bars embedded in concrete, the volume of corrosion products is approximately 2–4 times that of the original steel [13,14]. As a result, the corrosion products exert pressure on the surrounding concrete, which causes cracks at the interface between steel bars and concrete (IBSC) when the concrete strain reaches its ultimate tensile strain. Generally, the higher the strength of concrete, the larger the initial cracking time of IBSC under the same conditions [15]. As the development of steel corrosion, the cracks extend from the IBSC to the CCS, which provides a way for corrosive media to quickly reach the surface of steel bars, resulting in the accelerated corrosion of reinforcement, causing serious damage or even the spalling of the concrete cover [16,17]. Therefore, studying the corrosion-induced cracking process of the concrete cover is of great significance to predict the durability life of RC structures.

The models for studying the corrosion-induced cracking process of the concrete cover can be divided into three types including the empirical model, numerical model, and analytical model. The empirical models are derived by fitting the experimental data under different experimental conditions, which mainly consider the effects of some parameters on corrosion-induced cracking, such as concrete cover thickness, the ratio of concrete cover thickness to the diameter of steel bars, concrete strength, mass loss of steel bars, and so on [18,19,20]. Therefore, the applicability of these empirical models is limited to certain conditions, which cannot really reveal the corrosion-induced cracking mechanism of the concrete cover. The numerical models adopted by researchers are used to consider the non-linear behavior during the process of corrosion-induced cracking of the concrete cover, and the resulting equilibrium equations are solved numerically by either finite element [21,22,23] or finite difference methods [24]. Generally, the analytical models require some assumptions considering the material’s behaviour and can be solved using a closed-form solution, and in the analytical models, the parameters have specific physical meanings [1]. At present, different analytical models have been proposed to analyse the corrosion-induced cracking of the concrete cover based on the thick-walled cylinder model, in which the concrete cover is divided into an inside cracked and an outside un-cracked region [13,25,26,27,28].

The three-stage model [16] has been widely used to quantitatively describe the cracking process of the concrete cover caused by the corrosion of steel bars. In this model, it is assumed that the corrosion-induced cracking process of the concrete cover is divided into three stages: (1) the stage when corrosion products fill the pores at the IBSC, (2) the stage in which the corrosion products exert expansion pressure on the surrounding concrete, and (3) the stage in which the corrosion products fill the cracks. According to the three-stage model, the corrosion amount of steel bars corresponding to the three stages should be considered to predict the corrosion-induced cracking time of the concrete cover.

However, some researchers [29,30,31,32] have found that the average thickness of the rust-filling layer increased with the thickness of the rust layer until the thickness of the rust layer reached a critical value, that is, the rust was constantly filling the pores of surrounding concrete during the formation of rust layer, which is obviously different from the assumptions of the three-stage model. They also found that, before the corrosion-induced cracks reached the CCS, the rust would not fill the cracks and the pores of concrete near the cracks. Similarly, Chernin et al. [33] revealed that while the rust filled the pores of the IBSC, it continuously filled the pores of the surrounding concrete. Zhao et al. [34] also confirmed that when the concrete cover surface cracked induced by corrosion, the rust-filling layer was still developing. Thus, the rust filling the pores of the surrounding concrete should be taken into consideration when establishing the corrosion-induced cracking model of the concrete cover.

Because the rust fills the pores of the concrete, a rust-filling layer is formed in the surrounding concrete. Some researchers have established corrosion-induced cracking models of the concrete cover considering the effect of the rust-filling layer, while the effect of the load was not considered in their models [30,31]. During the service stage, RC structures suffer from different loads, and the loads affect the corrosion characteristics of steel bars embedded in concrete [35,36,37]. In contrast to that without considering load, the load can shorten the initial corrosion time, change the corrosion rate and the corrosion distribution, and cause local pitting of steel bars in RC structures [38,39,40], resulting in serious corrosion-induced cracking of the concrete cover [41,42]. Therefore, the influence of load should be considered when establishing the corrosion-induced cracking model of the concrete cover. However, to the best knowledge of the authors, the study of the corrosion-induced cracking model considering the effect of the load is severely inadequate.

To fill this research gap, in this paper, based on the thick-walled cylinder model, the effect of the load was considered to analyse the critical corrosion depth of steel bars when the IBSC cracked and the CCS cracked. Then, a prediction model for the corrosion-induced longitudinal cracking (CLC) time (i.e., initiation cracking time) of the CCS of RC structures under load was proposed, and the rationality of the prediction model was verified by the experimental data of Wang et al. [43]. Finally, based on the proposed prediction model, the influence of load on the CLC time of CCS was analysed.

## 2. Interaction between Ribbed Steel Bars and Surrounding Concrete under Load

**Figure 1:**
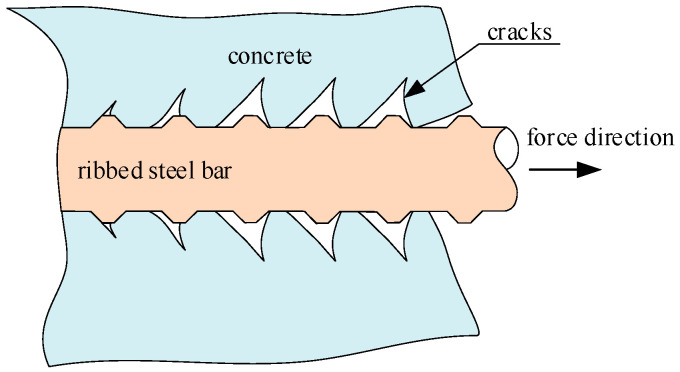
Distribution of cracks near the transverse ribs of ribbed steel bars under load.

The bonding force between ribbed steel bars and surrounding concrete consists of three parts: the chemical adhesive force between the cement gel and the surface of steel bars, the friction force between steel bars and concrete, and the mechanical interlocking force between the transverse ribs of steel bars and concrete. Among them, the mechanical interlocking force is the main part of the bonding force. The oblique extrusion force between the transverse ribs of steel bars and the surrounding concrete forms slip resistance. The component force of the oblique extrusion force along the axial direction of the steel bars makes the concrete between two transverse ribs bend and shear as a cantilever beam, which causes oblique cracks inside the concrete. The radial component of the oblique extrusion force makes the surrounding concrete like a pipe wall under internal pressure, resulting in hoop tensile stress inside the concrete, which causes radial cracks in the surrounding concrete. After the ribbed steel bars are subjected to a force, the distribution of cracks near the transverse ribs is shown in Figure 1.

**Figure 2:**
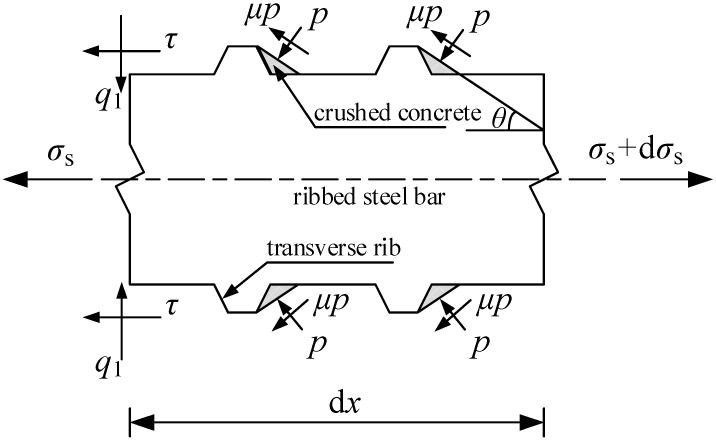
Interaction between the ribbed steel bars and the surrounding concrete under load.

After the ribbed steel bars are subjected to a force, the surrounding concrete produces oblique extrusion force *p* on transverse ribs, as shown in Figure 2. In the figure, *θ* is the angle between the concrete sliding surface near the transverse ribs and the axis of ribbed steel bars, and *μ* is the friction coefficient between the ribbed steel bars and concrete. By decomposing the oblique extrusion force *p* and friction force *μp* on the concrete sliding surface along the axis and radial directions of the ribbed steel bars, the bond stress *τ* and the radial pressure *q*_1_ can be obtained, as written in Equations (1) and (2), respectively.

(1) τ=psinθ+μpcosθ

(2) $$q_{1}=p\cos\theta -\mu p\sin\theta$$

Tepfers [44] found that the angle *θ* as shown in Figure 2 was 30°–40°, and Xu [45] found that the angle ranged from 10° to 40°, with an average of 25°. The friction coefficient *μ* between the ribbed steel bars and concrete increases with the corrosion degree of ribbed steel bars, and if the ribbed steel bars are not corroded, the friction coefficient can be taken as 0.3 [45]. Guo and Shi [46] showed that the steel bars changed from a non-corrosion state to a corrosion state, and the variation range of the friction coefficient was 0.2–0.6. Zhao and Xiao [47] confirmed that the friction coefficient between the concrete and rolled steel was 0.25–0.35. According to the above research results, *θ* is taken as 30° and *μ* is taken as 0.35 in this paper.

The relationship between the bond stress *τ* and the radial pressure *q*_1_ can be derived from Equations (1) and (2):

(3) $$q_{1}=\frac{\cos\theta -\mu \sin\theta }{\sin\theta +\mu \cos\theta }\tau$$

The bond stress as shown in Figure 2 can be determined by establishing the mechanical equilibrium equation along the axis direction of the ribbed steel bars:

(4) $$\tau =\frac{A_{s}}{2\pi R}\frac{d\sigma_{s}}{dx}=\frac{R}{2}\frac{d\sigma_{s}}{dx}$$

where *R* is the radius of the ribbed steel bars, and *σ*_s_ is the tensile stress of the ribbed steel bars.

Substituting Equation (4) into Equation (3), the radial pressure can be obtained as:

(5) $$q_{1}=\frac{\cos\theta -\mu \sin\theta }{\sin\theta +\mu \cos\theta }\frac{R}{2}\frac{d\sigma_{s}}{dx}$$

**Figure 3:**
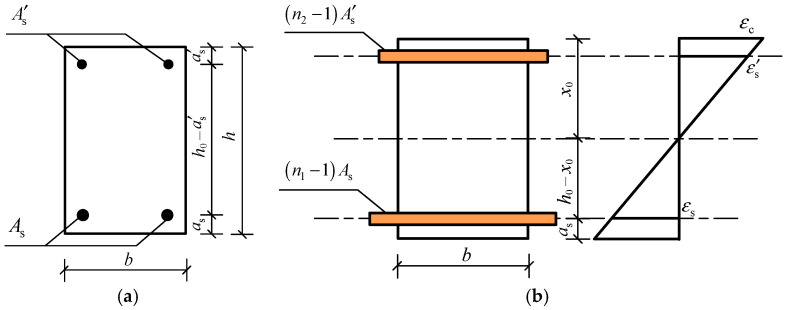
Transformed cross-section before concrete cracking. (**a**) Original cross-section; (**b**) transformed cross-section.

To calculate d*σ*_s_/d*x* in Equation (5), it is first necessary to derive the equation between the tensile stress of the ribbed steel bars and the bending moment, and then take the derivative of *x* on both sides of the equation to obtain d*σ*_s_/d*x*. The detailed calculation process is as follows:

(1) During the service stage, before the load-induced cracking of RC members, the full cross-section of RC members is subjected to force. When calculating the tensile stress of ribbed steel bars under the bending moment, the cross-section of the steel bars needs to be transformed to the concrete section. For RC beams with a rectangular cross-section, the transformed cross-section is shown in Figure 3, and the area of the transformed cross-section can be calculated by Equation (6).

(6) $$A_{0}=bh+\left(n_{1}-1\right)A_{s}+\left(n_{2}-1\right){A^{\prime }}_{s}$$

where *A*_0_ is the area of cross-section after transformation, *n*_1_ denotes the ratio of the elastic modulus of tensile steel bars to that of concrete, *n*_2_ refers to the ratio of the elastic modulus of the compressive steel bars to that of concrete, *A*_s_ is the area of cross-section of tensile steel bars, ${A^{\prime }}_{s}^{}$ is the area of cross-section of compressive steel bars, and *b* and *h* are the width and height of the cross-section, respectively.

The height of the compression zone of the transformed cross-section can be determined by the equal area moment of the tension zone and compression zone to the neutral axis of the cross-section:

(7) $$x_{0}=\frac{\frac{1}{2}bh^{2}+\left(n_{1}-1\right)A_{s}h_{0}+\left(n_{2}-1\right){A^{\prime }}_{s}{a^{\prime }}_{s}}{bh+\left(n_{1}-1\right)A_{s}+\left(n_{2}-1\right){A^{\prime }}_{s}}$$

where *x*_0_ is the height of the compression zone, and *h*_0_ is the effective height of the cross-section.

After the height of the compression zone is obtained, the moment of inertia *I*_0_ of the transformed cross-section can be expressed as:

(8) $$I_{0}=\frac{b}{3}\left[x_{0}^{3}+{\left(h-x_{0}\right)}^{3}\right]+\left(n_{1}-1\right)A_{s}{\left(h_{0}-x_{0}\right)}^{2}+\left(n_{2}-1\right){A^{\prime }}_{s}{\left(x_{0}-{a^{\prime }}_{s}\right)}^{2}$$

Therefore, before load-induced cracking of RC beams, the tensile stress of ribbed steel bars under bending moment is:

(9) $$\sigma_{s}=\frac{n_{1}M\left(h_{0}-x_{0}\right)}{I_{0}}$$

where *M* is the bending moment.

During the service stage, after the load-induced cracking of RC beams, the concrete in the tension zone of the cross-section exits work, and only the steel bars in the tension zone are subjected to force. For RC beams with a rectangular cross-section, the tensile stress of steel bars in the tension zone of the cross-section can be calculated according to the method of the transformed cross-section, or calculated according to the method stipulated in China Standard (GB 50010) [48]. The latter method is adopted in this paper, as written in Equation (10).

(10) $$\sigma_{s}=\frac{M}{0.87A_{s}h_{0}}$$

**Figure 4:**
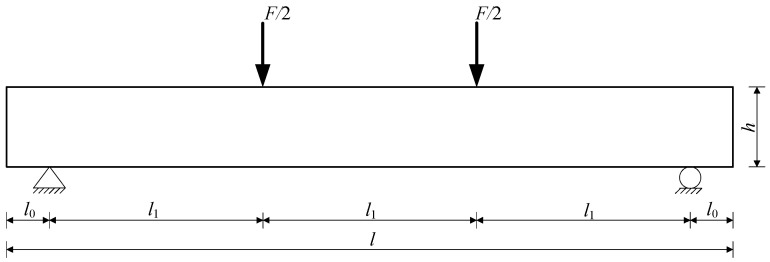
RC beams under the third-point concentrated load.

(2) For the RC beams with a rectangular cross-section, the bending moment is often different along the length of beams under load. Taking the RC beams subjected to third-point concentrated load (i.e., F/2) as an example (Figure 4), the bending moment can be calculated according to Equation (11).

(11) $$M\left(x\right)=\{\begin{array}{l} \frac{F}{2}x,\;0\leq x\leq l_{1}\\ \frac{F}{2}l_{1},\;l_{1}\leq x\leq 2l_{1}\\ \frac{F}{2}\left(3l_{1}-x\right),\;2l_{1}\leq x\leq 3l_{1} \end{array}$$

where *M*(*x*) is the bending moment at the distance *x* from the left support of beams under the third-point concentrated load, and *l*_1_ is 1/3 of the calculated span of RC beams.

Substituting Equation (11) into Equations (9) and (10), respectively, the tensile stress of ribbed steel bars can be obtained, and then Equation (5) can be rewritten as:

Before load-induced cracking of RC beams:

(12) $$q_{1}=\{\begin{array}{l} \frac{n_{1}F\left(h_{0}-x_{0}\right)}{2I_{0}}\frac{\cos\theta -\mu \sin\theta }{\sin\theta +\mu \cos\theta }\frac{R}{2},\;0\leq x\leq l_{1}\\ 0,\;l_{1}\leq x\leq 2l_{1}\\ -\frac{n_{1}F\left(h_{0}-x_{0}\right)}{2I_{0}}\frac{\cos\theta -\mu \sin\theta }{\sin\theta +\mu \cos\theta }\frac{R}{2},\;2l_{1}\leq x\leq 3l_{1} \end{array}$$

After load-induced cracking of RC beams:

(13) $$q_{1}=\{\begin{array}{l} \frac{F}{1.74A_{s}h_{0}}\frac{\cos\theta -\mu \sin\theta }{\sin\theta +\mu \cos\theta }\frac{R}{2},\;0\leq x\leq l_{1}\\ 0,\;l_{1}\leq x\leq 2l_{1}\\ -\frac{F}{1.74A_{s}h_{0}}\frac{\cos\theta -\mu \sin\theta }{\sin\theta +\mu \cos\theta }\frac{R}{2},\;2l_{1}\leq x\leq 3l_{1} \end{array}$$

This paper only gives the calculation formulas of radial pressure when the RC beams with rectangular cross-sections are subjected to third-point concentrated loads. Similarly, the calculation method of radial pressure can be established for beams under other types of loads.

**Figure 5:**
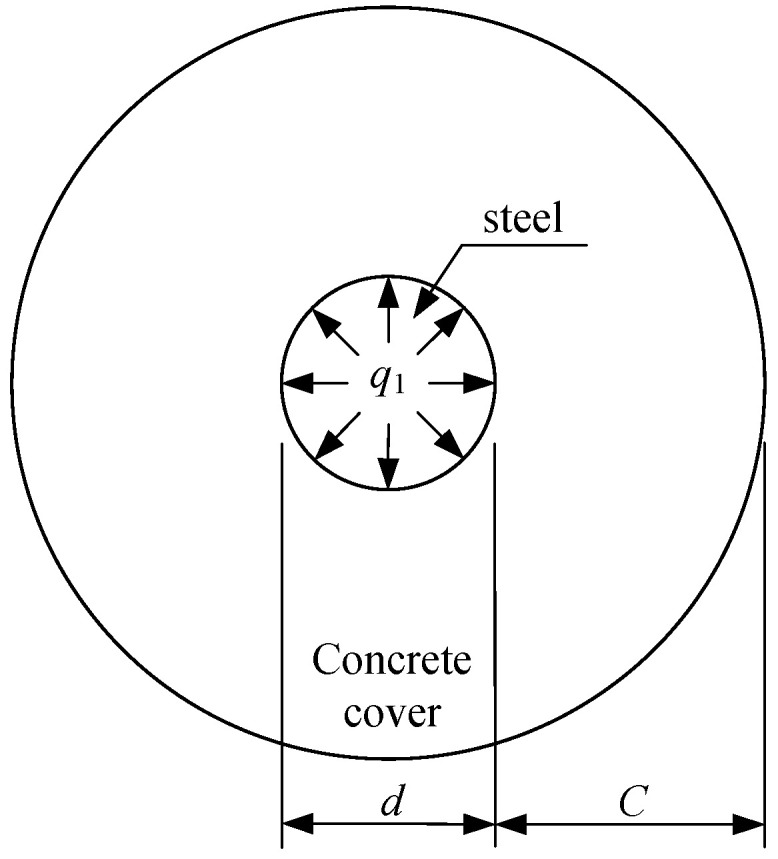
Thick-walled cylinder model.

For RC structures under load, before the corrosion of steel bars, there is only radial pressure between steel bars and surrounding concrete. The thick-walled cylinder model [44,49,50] is usually adopted to calculate the interaction between steel bars and the concrete cover, as shown in Figure 5. In this figure, *C* is the thickness of the concrete cover, *d* is the diameter of steel bars, and *q*_1_ is the radial pressure.

Along the longitudinal direction of the steel bars, the concrete cover may be in uncracked, partially cracked, or completely cracked states under radial pressure. This paper only discusses the longitudinal cracking of the concrete cover under the coupled effects of load and steel corrosion; hence, the transverse cracking of the concrete cover is not considered and is out of the scope of this paper. Assuming that the longitudinal cracks do not generate in concrete cover under radial pressure, that is, the concrete cover is in the elastic deformation stage (Figure 5). Therefore, the elastic theory can be adopted to calculate the stress distribution in the concrete cover under radial pressure. According to the theory of elasticity [51], the hoop stress at any position *r*_x_ (*R* ≤ *r*_x_ ≤ *b*) of the thick-walled cylinder under radial pressure can be expressed as:

(14) $$\sigma_{\theta }\left(r_{x}\right)=\frac{q_{1}R^{2}}{b^{2}-R^{2}}\left(1+\frac{b^{2}}{r_{x}^{2}}\right)$$

where *b* is the outer radius of the thick-walled cylinder, *b* = *d*/2 + *C*.

Equation (14) can be used to judge the longitudinal cracking of the concrete cover: if *σ*_θ_(*R*) < *f*_t_, no cracking, and if *σ*_θ_(*R*) ≥ *f*_t_ and *σ*_θ_(*b*) < *f*_t_, partial cracking. After the corrosion of the steel bars, the surrounding concrete does not crack immediately. Corrosion products accumulate at the IBSC and continue to exert corrosion expansion pressure *q*_2_ on the surrounding concrete. Therefore, the time when longitudinal cracks appear on the CCS under the combined action of corrosion expansion pressure *q*_2_ and radial pressure *q*_1_ is the longitudinal cracking time of CCS under the coupled effects of load and steel corrosion. The total radial pressure *q* calculated by Equation (15) can be directly applied to the inner wall of the thick-walled cylinder, and then the elasticity and damage mechanics theories are adopted to derive the time for the appearance of longitudinal cracks on the CCS.

(15) $$q=q_{1}+q_{2}$$

## 3. Rust Filling Model

**Figure 6:**
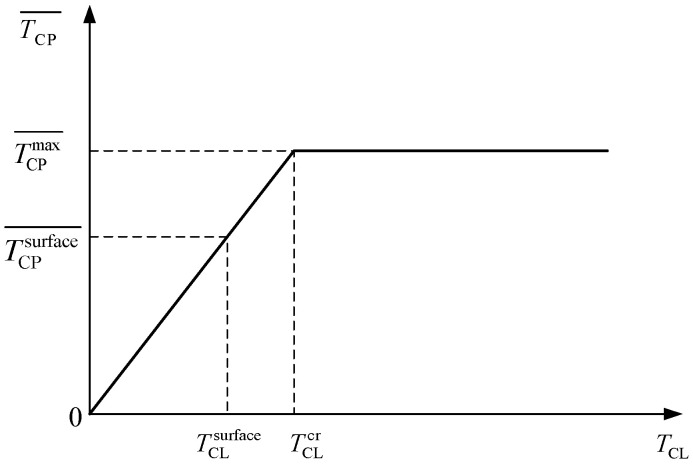
Relationship between the average thickness of the rust-filling layer and the thickness of the rust layer.

During the corrosion-induced cracking process of the concrete cover, corrosion products accumulate at the IBSC forming the rust layer and filling the pores of surrounding concrete forming the rust-filling layer. The process of corrosion products filling the pores of surrounding concrete is affected by many factors, such as the position of the aggregate and the distribution of pores. Zhao et al. [29,30,32] and Wu [31] found that the average thickness of the rust-filling layer increased with the thickness of the rust layer until the thickness of the rust layer reached a critical value, that is, the corrosion products were constantly filling the pores of surrounding concrete during the formation of the rust layer. The relationship between the average thickness of the rust-filling layer and the thickness of the rust layer is shown in Figure 6 and can be expressed by Equation (16) [29,30].

(16) $$\{\begin{array}{l} \bar{T_{\mathrm{CP}}}=k_{T}\times T_{\mathrm{CL}},\;T_{\mathrm{CL}}<T_{\mathrm{CL}}^{\mathrm{cr}}\\ \bar{T_{\mathrm{CP}}}=\bar{T_{\mathrm{CP}}^{\max}}=k_{T}\times T_{\mathrm{CL}}^{\mathrm{cr}},\;T_{\mathrm{CL}}\geq T_{\mathrm{CL}}^{\mathrm{cr}} \end{array}$$

where *T*_CL_ is the thickness of the rust layer at the IBSC, $\bar{T_{\mathrm{CP}}}$ denotes the average thickness of the rust-filling layer; $\bar{T_{\mathrm{CP}}^{\max}}$ is the maximum value of $\bar{T_{\mathrm{CP}}}$, $T_{\mathrm{CL}}^{\mathrm{cr}}$ is the $T_{\mathrm{CL}}$ corresponding to $\bar{T_{\mathrm{CP}}^{\max}}$, and *k*_T_ is the ratio of $\bar{T_{\mathrm{CP}}^{\max}}$ to $T_{\mathrm{CL}}^{\mathrm{cr}}$.

**Figure 7:**
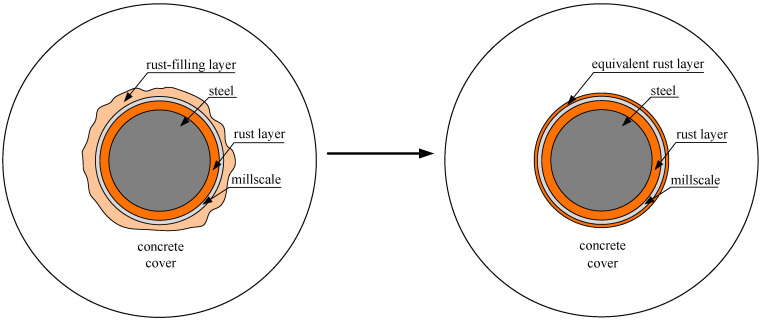
The rust-filling layer is converted to an equivalent rust layer.

To consider the influence of the rust-filling layer in the corrosion-induced cracking model of the concrete cover, the rust-filling layer should be converted to the equivalent rust layer as shown in Figure 7, and the thickness of the equivalent rust layer can be expressed as [30]:

(17) $$T_{\mathrm{CL},\mathrm{pore}}=\frac{\gamma \times \int_{0}^{2\pi }T_{\mathrm{CP}}Rd\theta }{2\pi R}$$

where *T*_CL,pore_ is the thickness of the equivalent rust layer, *T*_CP_ denotes the thickness of the rust-filling layer, and *γ* is the porosity of concrete around the steel bars, which can be approximately calculated as [30]:

(18) $$\gamma =\frac{W/C-0.36\alpha }{W/C+0.32}$$

where *W*/*C* is the water–cement ratio of concrete, and *α* is the hydration degree of concrete.

Generally, the average thickness of the rust-filling layer does not reach the maximum average thickness before the CCS cracks [30,31], as shown in Figure 6. Therefore, when establishing a prediction model for the cracking of CCS, only the case of $T_{\mathrm{CL}}$ < $T_{\mathrm{CL}}^{\mathrm{cr}}$ in Equation (16) is considered. The thickness of the equivalent rust layer can be determined according to Equation (17):

(19) $$T_{\mathrm{CL},\mathrm{pore}}=\gamma \times \bar{T_{\mathrm{CP}}}=\gamma \times k_{T}\times T_{\mathrm{CL}},\;T_{\mathrm{CL}}<T_{\mathrm{CL}}^{\mathrm{cr}}$$

It can be found from Equation (19) that the thickness of the equivalent rust layer is related to the type of concrete. After the corrosion of the steel bars, assuming that *V*_rust_ is the total volume of rust, and *V*_steel_ is the volume lost by the steel bars due to corrosion, the actual rust volume expansion ratio *n* can be defined as [30]:

(20) $$n=\frac{V_{\mathrm{rust}}}{V_{\mathrm{steel}}}$$

The total volume of rust *V*_rust_ is the sum of the volume of rust filling the concrete pores *V*_rust,CP_ and the volume of rust layer *V*_rust,CL_:

(21) $$V_{\mathrm{rust}}=V_{\mathrm{rust},\mathrm{CP}}+V_{\mathrm{rust},\mathrm{CL}}$$

However, during the corrosion process of steel bars, only the rust layer exerts extrusion force on the surrounding concrete, which causes the concrete cover to crack. Therefore, the nominal rust volume expansion ratio *n*_0_ can be defined as [30]:

(22) $$n_{0}=\frac{V_{\mathrm{rust},\mathrm{CL}}}{V_{\mathrm{steel}}}$$

The relationship between the actual rust volume expansion ratio and the nominal rust volume expansion ratio is [30]:

(23) $$\frac{n}{n_{0}}=\frac{V_{\mathrm{rust}}/V_{\mathrm{steel}}}{V_{\mathrm{rust},\mathrm{CL}}/V_{\mathrm{steel}}}=\frac{V_{\mathrm{rust},\mathrm{CL}}+V_{\mathrm{rust},\mathrm{CP}}}{V_{\mathrm{rust},\mathrm{CL}}}=1+\frac{V_{\mathrm{rust},\mathrm{CP}}}{V_{\mathrm{rust},\mathrm{CL}}}\approx 1+\frac{T_{\mathrm{CL},\mathrm{pore}}}{T_{\mathrm{CL}}}$$

The following equation can be derived from Equations (19) and (23):

(24) $$n_{0}=\frac{n}{1+\gamma \times k_{T}}$$

Equation (24) shows that when *n* = *n*_0_, the volume of rust filled into the pores of the concrete around the steel bars is zero, and *n*_0_/*n* decreases with the increase of concrete porosity *γ*, that is, the volume of rust filled into the pores of concrete around the steel bars increases with concrete porosity. It can be seen from the above analysis that *n*_0_ should be used instead of *n* when establishing the corrosion-induced cracking model of the concrete cover. However, for different types of concrete, before calculating *n*_0_, the values of *γ* and *k*_T_ need to be determined.

## 4. Non-cracking Stage Model of Concrete Cover

**Figure 8:**
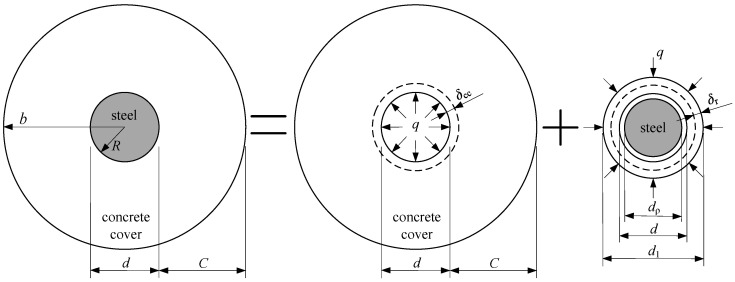
Radial deformations of concrete and rust layer at the IBSC under the total radial pressure in the non-cracking stage of the concrete cover.

For the thick-walled cylinder model, as shown in Figure 8, when the IBSC does not crack under the total radial pressure *q*, the stress and strain at any point in the concrete cover can be calculated by the elastic theory. In Figure 8, *δ*_cc_ and *δ*_r_ are, respectively, the radial deformations of the concrete and rust layers at the IBSC, *d*_ρ_ is the residual diameter of the steel bars after corrosion, and *d*_1_ denotes the diameter of the steel bars after the free expansion of rust.

Stress components at any point in the concrete cover under the total radial pressure can be formulated as [13,30]:

(25) $$\{\begin{array}{l} \sigma_{r}=\frac{qR^{2}}{b^{2}-R^{2}}\left(1-\frac{b^{2}}{r_{x}^{2}}\right)\\ \sigma_{\theta }=\frac{qR^{2}}{b^{2}-R^{2}}\left(1+\frac{b^{2}}{r_{x}^{2}}\right) \end{array}$$

where *σ*_r_ and *σ*_θ_ are the radial stress and the hoop stress in the concrete cover at a distance *r*_x_ from the center of the steel bar, respectively, and *b* = *R* + *C.*

Strain components at any point in the concrete cover under the total radial pressure can be derived as [13,30]:

(26) $$\{\begin{array}{l} \varepsilon_{r}=\frac{1+\nu_{c}}{E_{c}}\frac{qR^{2}}{b^{2}-R^{2}}\left(1-2\nu_{c}-\frac{b^{2}}{r_{x}^{2}}\right)\\ \varepsilon_{\theta }=\frac{1+\nu_{c}}{E_{c}}\frac{qR^{2}}{b^{2}-R^{2}}\left(1-2\nu_{c}+\frac{b^{2}}{r_{x}^{2}}\right) \end{array}$$

where *ε*_r_ and *ε*_θ_ are the radial strain and the hoop strain in concrete cover at the distance *r*_x_ from the center of the steel bar, respectively, and *E*_c_ and *v*_c_ are the elastic modulus and Poisson’s ratio of concrete, respectively.

According to the elastic theory, the radial displacement *u*_r_ at any point in the concrete cover can be derived by *ε*_θ_ = *u*_r_/*r*_x_:

(27) $$u_{r}=\frac{1+\nu_{c}}{E_{c}}\frac{qR^{2}}{b^{2}-R^{2}}\left(1-2\nu_{c}+\frac{b^{2}}{r_{x}^{2}}\right)r_{x}$$

The critical state of cracking at the IBSC is defined as the hoop strain of concrete reaching ultimate tensile strain:

(28) $$\{\begin{array}{l} \varepsilon_{\theta }=\frac{u_{r}}{r_{x}}\\ \varepsilon_{\theta |r_{x}=R}=\varepsilon_{t}=\frac{f_{t}}{E_{c}} \end{array}$$

where *ε*_t_ and *f*_t_ are the ultimate tensile strain and tensile strength of concrete, respectively.

When the IBSC cracks, the critical value *q*^inner^ of total radial pressure *q* can be obtained on the basis of Equations (27) and (28):

(29) $$q^{\mathrm{inner}}=\frac{E_{c}\varepsilon_{t}\left(b^{2}-R^{2}\right)}{\left(1+\nu_{c}\right)\left(1-2\nu_{c}+b^{2}/R^{2}\right)R^{2}}$$

According to the deformation coordination conditions of the concrete and rust layer at the IBSC, the following equation can be obtained [13,30]:

(30) $$R+\delta_{\mathrm{cc}}=R_{1}+\delta_{r}$$

where *R*_1_ is the radius of steel bars after the free expansion of rust.

The calculation methods of parameters in Equation (30) are [13,30]:

(31) $$\{\begin{array}{l} R_{1}=R\sqrt{\left(n_{0}-1\right)\eta_{s}+1}\\ \delta_{\mathrm{cc}}=u_{r|r_{x}=R}=\frac{1+\nu_{c}}{E_{c}}\frac{qR^{2}}{b^{2}-R^{2}}\left(1-2\nu_{c}+\frac{b^{2}}{R^{2}}\right)R\\ \delta_{r}=-\frac{n_{0}\left(1-\nu_{r}^{2}\right)R\sqrt{\left(n_{0}-1\right)\eta_{s}+1}}{E_{r}\left\{\left[\left(1+v_{r}\right)n_{0}-2\right]+2/\eta_{s}\right\}}q \end{array}$$

where *η*_s_ is the mass loss of steel bars, *E*_r_ is the elastic modulus of rust, which can be taken as 100 MPa [52], and *v*_r_ is the Poisson’s ratio of rust, which can be taken as 0.25 [52].

When the IBSC cracks, the relationship between the critical total radial pressure *q*^inner^ and critical mass loss $\eta_{s}^{\mathrm{inner}}$ of steel bars can be derived from Equations (29) to (31):

(32) $$R+\frac{1+\nu_{c}}{E_{c}}\frac{q^{\mathrm{inner}}R^{2}}{b^{2}-R^{2}}\left(1-2\nu_{c}+\frac{b^{2}}{R^{2}}\right)R=R\sqrt{\left(n_{0}-1\right)\eta_{s}^{\mathrm{inner}}+1}-\frac{n_{0}\left(1-\nu_{r}^{2}\right)R\sqrt{\left(n_{0}-1\right)\eta_{s}^{\mathrm{inner}}+1}}{E_{r}\left\{\left[\left(1+v_{r}\right)n_{0}-2\right]+2/\eta_{s}^{\mathrm{inner}}\right\}}q^{\mathrm{inner}}$$

The critical total radial pressure *q*^inner^ can be derived from Equation (32):

(33) $$q^{\mathrm{inner}}=\frac{\sqrt{\left(n_{0}-1\right)\eta_{s}^{\mathrm{inner}}+1}-1}{\frac{\left(1+v_{c}\right){\left(R+C\right)}^{2}+\left(1-2v_{c}\right)R^{2}}{E_{c}\left(2RC+C^{2}\right)}+\frac{n_{0}\left(1-\nu_{r}^{2}\right)\sqrt{\left(n_{0}-1\right)\eta_{s}^{\mathrm{inner}}+1}}{E_{r}\left\{\left[\left(1+v_{r}\right)n_{0}-2\right]+2/\eta_{s}^{\mathrm{inner}}\right\}}}$$

Substituting $x_{1}=\sqrt{\left(n_{0}-1\right)\eta_{s}^{\mathrm{inner}}+1}$ into Equation (33), and assuming *M*_1_= [(1 + *v*_c_)(*R* + *C*)^2^ + (1 − 2*v*_c_)*R*^2^]/[*E*_c_(2*RC* + *C*^2^)], *M*_2_ = *n*_0_(1 − $v_{r}^{2}$)/*E*_r_, *M*_3_ = (1 + *v*_c_)*n*_0_ − 2, the following equations can be obtained:

(34a) $$\alpha_{11}x_{1}^{3}+\alpha_{22}x_{1}^{2}+\alpha_{33}x_{1}+\alpha_{44}=0$$

(34b) $$\{\begin{array}{l} \alpha_{11}=M_{2}-M_{3}/q^{\mathrm{inner}}\\ \alpha_{22}=M_{3}/q^{\mathrm{inner}}+M_{1}M_{3}\\ \alpha_{33}=M_{3}/q^{\mathrm{inner}}-M_{2}-2\left(n_{0}-1\right)/q^{\mathrm{inner}}\\ \alpha_{44}=2M_{1}\left(n_{0}-1\right)+2\left(n_{0}-1\right)/q^{\mathrm{inner}}-M_{1}M_{3}-M_{3}/q^{\mathrm{inner}} \end{array}$$

The algebraic cubic equation as shown in Equation (34a) has three roots. However, only one analytic real root for Equation (34a) is useful, which can be calculated as [53]:

(35a) $$x_{1}=\sqrt[{3}]{-\frac{N_{2}}{2}+\sqrt{{\left(\frac{N_{2}}{2}\right)}^{2}+{\left(\frac{N_{1}}{3}\right)}^{3}}}+\sqrt[{3}]{-\frac{N_{2}}{2}-\sqrt{{\left(\frac{N_{2}}{2}\right)}^{2}+{\left(\frac{N_{1}}{3}\right)}^{3}}}-\frac{\alpha_{22}}{3\alpha_{11}}$$

(35b) $$N_{1}=\frac{\alpha_{33}}{\alpha_{11}}-\frac{1}{3}{\left(\frac{\alpha_{22}}{\alpha_{11}}\right)}^{2}$$

(35c) $$N_{2}=\frac{2}{27}{\left(\frac{\alpha_{22}}{\alpha_{11}}\right)}^{3}-\frac{1}{3}\left(\frac{\alpha_{22}}{\alpha_{11}}\right)\left(\frac{\alpha_{33}}{\alpha_{11}}\right)+\frac{\alpha_{44}}{\alpha_{11}}$$

If Equation (34a) has no real roots, a real root can be approximately obtained as the value of *x*_1_ through the MATLAB program, and then the critical mass loss of steel bars can be determined as:

(36) $$\eta_{s}^{\mathrm{inner}}=\frac{x_{1}^{2}-1}{n_{0}-1}$$

Through the critical mass loss of steel bars, the critical corrosion depth of steel bars can be derived as:

(37a) $$\delta_{\mathrm{steel}}^{\mathrm{inner}}=\frac{d-d_{\rho }}{2}$$

(37b) $$d_{\rho }=\sqrt{1-\eta_{s}^{\mathrm{inner}}}d$$

where $\delta_{\mathrm{steel}}^{\mathrm{inner}}$ is the critical corrosion depth of steel bars when the IBSC cracks.

According to the calculation model of the critical corrosion depth of steel bars established above, to analyse the influence of load on the critical corrosion depth of steel bars when the IBSC cracks, an example is adopted. Assuming that the length of an RC beam is 2300 mm, its calculated length is 2100 mm, the size of its cross-section is 150 (width) × 250 (height) mm, the strength grade of concrete is C30, the tensile steel bars are two HRB335 ribbed steel bars with a diameter of *d* = 18 mm and the yield strength is *f*_y_ = 335 MPa, the thickness of the concrete cover is *C* = 30 mm, and the actual rust volume expansion ratio is *n* = 2. The parameters of concrete are tensile strength *f*_t_ = 2.01 MPa, compressive strength *f*_c_ = 20.1 MPa, elastic modulus *E*_c_ = 30 GPa, and Poisson’s ratio *v*_c_ = 0.2. The range of *n*/*n*_0_ is about 1–1.2 [31], and 1.0 and 1.2 are taken in this example.

**Figure 9:**
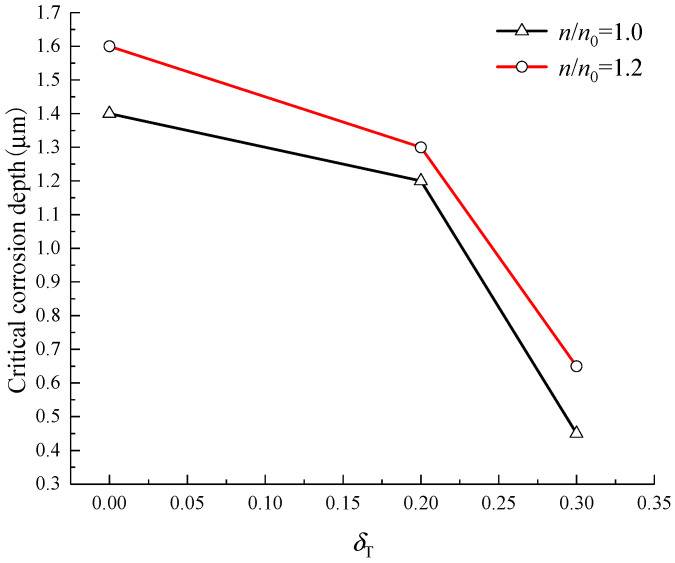
Critical corrosion depth of steel bars when the IBSC cracks.

For the RC beam under a third-point concentrated load, the radial pressure *q*_1_ occurs at the IBSC, which causes hoop tensile stress in the concrete cover, as shown in Equation (14). Assuming that the hoop tensile stress at the position *r*_x_ = *R* of the concrete cover caused by the radial pressure is 0, 0.2, and 0.3 times the tensile strength of concrete, the tensile stress level *δ*_T_ = 0, 0.2, and 0.3, meanwhile, the IBSC does not crack [54]. Figure 9 shows the critical corrosion depth of steel bars when the IBSC cracks are induced by corrosion under load. It can be seen from Figure 9 that the load has a significant effect on the critical corrosion depth of the steel bars. The greater the load, the smaller the critical corrosion depth, and for the two cases of *n/n*_0_ = 1.0 and *n/n*_0_ = 1.2, when the tensile stress level *δ*_T_ increases from 0 to 0.3, the critical corrosion depth is reduced by 67.9% and 59.4%, respectively. Therefore, the influence of load needs to be considered when establishing the corrosion-induced cracking model of the concrete cover. Figure 9 also depicts that when *n/n*_0_ = 1.2, the critical corrosion depth is significantly larger than that of *n/n*_0_ = 1.0, which is because, the larger the *n/n*_0_, the more rust filled into the pores of concrete around steel bars, and the rust-filling layer does not produce squeezing force on the IBSC. As a result, the more serious the corrosion of steel bars, the larger the critical corrosion depth of steel bars.

## 5. Partial Cracking Stage Model of Concrete Cover

**Figure 10:**
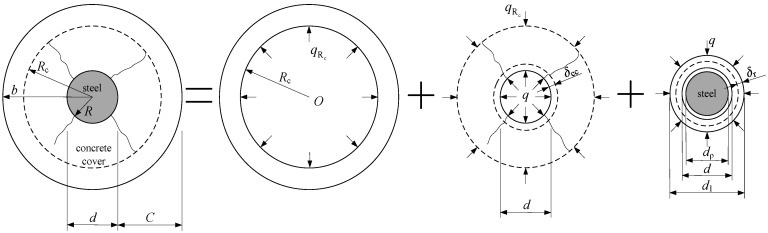
Radial deformations of concrete and rust layers at the IBSC under the total radial pressure in the partial cracking stage of the concrete cover.

After the IBSC cracks under the total radial pressure, the cracks propagate radially to the surface of the cylinder. Therefore, the thick-walled cylinder can be divided into two coaxial cylinders. The inside one is a cracked cylinder and the outside one is an intact cylinder, as depicted in Figure 10. In the figure, *R*_c_ represents the radius of the interface between the cracked cylinder and the intact cylinder.

The cracked concrete inside the thick-walled cylinder is assumed to be non-uniform orthotropic linear elastic material, and the outside intact concrete is assumed to be isotropic linear elastic material [33]. Then, the stress component and strain component at any point in the intact concrete can be derived from elastic theory [55]:

Stress components:

(38) $$\{\begin{array}{l} \sigma_{r}=\frac{q_{R_{c}}R_{c}^{2}}{b^{2}-R_{c}^{2}}\left(1-\frac{b^{2}}{r_{x}^{2}}\right)\\ \sigma_{\theta }=\frac{q_{R_{c}}R_{c}^{2}}{b^{2}-R_{c}^{2}}\left(1+\frac{b^{2}}{r_{x}^{2}}\right) \end{array}$$

where $q_{R_{c}}$ is the radial pressure at the interface between cracked and intact cylinders.

Strain components:

(39) $$\{\begin{array}{l} \varepsilon_{r}=\frac{1+\nu_{c}}{E_{c}}\frac{q_{R_{c}}R_{c}^{2}}{b^{2}-R_{c}^{2}}\left(1-2\nu_{c}-\frac{b^{2}}{r_{x}^{2}}\right)\\ \varepsilon_{\theta }=\frac{1+\nu_{c}}{E_{c}}\frac{q_{R_{c}}R_{c}^{2}}{b^{2}-R_{c}^{2}}\left(1-2\nu_{c}+\frac{b^{2}}{r_{x}^{2}}\right) \end{array}$$

For the cracked cylinder, assuming that the cracks are uniformly distributed on the circumference of the cracked cylinder and propagate outwards radially, the damage degree of concrete along the direction of cracks is different, which near the steel bars is larger than that of concrete far away from the steel bars. The relationship between the radial stress and hoop stress at any point in the cracked cylinder is [55]:

(40) $$\frac{d\sigma_{r}}{dr_{x}}+\frac{\sigma_{r}-\sigma_{\theta }}{r_{x}}=0$$

Based on the Mohr–Coulomb failure criterion [13] and Mazar’s damage model [56], the damage variable *D* is adopted to describe the damage degree of the cracked cylinder along the radial direction. Equation (40) can be rewritten as [57]:

(41) $$\sigma_{\theta }=\sigma_{r}\frac{1-\left(1-D\right)\sin\varphi }{1+\left(1-D\right)\sin\varphi }+\frac{2c\cos\varphi }{1+\left(1-D\right)\sin\varphi }$$

where *φ* is the internal friction angle of concrete, and *c* is the cohesive strength of concrete.

The internal friction angle of concrete decreases slightly with increasing concrete strength, which can be approximately taken as 37°, and the cohesive strength of concrete can be determined as [30,58]:

(42) $$c=\frac{\left(1-\sin\varphi \right)f_{c}}{2\cos\varphi }$$

where *f*_c_ is the compressive strength of concrete.

The damage variable *D* can be derived by Mazar’s damage equation [59]:

(43) $$D=1-\frac{\varepsilon_{t}\left(1-A_{t}\right)}{\varepsilon_{\theta }}-\frac{A_{t}}{\exp\left[B_{t}\left(\varepsilon_{\theta }-\varepsilon_{t}\right)\right]}$$

where *A*_t_ and *B*_t_ are the coefficients of Mazar’s damage model, 0.7 < *A*_t_ < 1 and 10^4^ < *B*_t_ < 10^5^.

Substituting Equation (41) into Equation (40) yields [13,30]:

(44) $$\sigma_{r}=\exp\left(-\int_{R}^{r_{x}}\frac{2\left(1-D\right)\sin\varphi }{\left[1+\left(1-D\right)\sin\varphi \right]x}dx\right)\left(\int_{R}^{r_{x}}\frac{2c\cos\varphi }{\left[1+\left(1-D\right)\sin\varphi \right]\xi }\exp\left(\int_{R}^{\xi }\frac{2\left(1-D\right)\sin\varphi }{\left[1+\left(1-D\right)\sin\varphi \right]x}dx\right)d\xi +C_{0}\right)$$

The boundary conditions are expressed as:

(45a) $$\sigma_{r}=-q,\;r_{x}=R$$

(45b) $$\{\begin{array}{l} D=0\\ \sigma_{r}=-q_{R_{c}},\;r_{x}=R_{c}\\ \varepsilon_{\theta }=\varepsilon_{t} \end{array}$$

Substituting Equation (45a) into Equation (44) yields:

(46) $$C_{0}=-q$$

Substituting Equation (45b) into Equation (39) results in:

(47) $$q_{R_{c}}=\frac{f_{t}}{1+\nu_{c}}\frac{b^{2}-R_{c}^{2}}{R_{c}^{2}}\frac{1}{1-2\nu_{c}+b^{2}/R_{c}^{2}}$$

Substituting Equation (45b) and Equation (46) into Equation (44), the total radial pressure can be derived as [13,30]:

(48) $$q=q_{R_{c}}{\left(\frac{R_{c}}{R}\right)}^{\frac{2\sin\varphi }{1+\sin\varphi }}+\frac{c\cos\varphi }{\sin\varphi }\left[{\left(\frac{R_{c}}{R}\right)}^{\frac{2\sin\varphi }{1+\sin\varphi }}-1\right]$$

**Figure 11:**
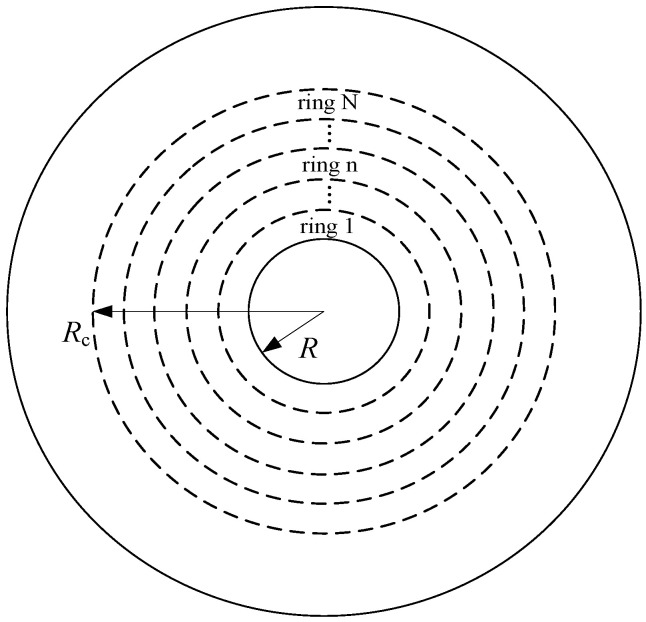
Partitions of the cracked part of the concrete cover.

Equation (48) can be simplified to Equation (29) when *R*_c_ = *R*. Hence, the calculated results obtained at the non-cracking stage and partial cracking stage of the concrete cover are continuous. To simplify the calculation, the cracked cylinder is divided into *N* concentric rings of equal thickness, as depicted in Figure 11. For each ring, the damage variable *D* can be considered constant. The radial displacement *u*_r_ in the cracked cylinder should satisfy the following equation [30,60,61]:

(49) $$\frac{d^{2}u_{r}}{dr_{x}^{2}}+\frac{1}{r_{x}}\frac{du_{r}}{dr_{x}}-\left(1-D\right)\frac{u_{r}}{r_{x}^{2}}=0$$

The radial displacement at any point in the cracked cylinder can be obtained from Equation (49), which is expressed as [13,30]:

(50) $$u_{r}=C_{1}r_{x}^{\sqrt{1-D}}+C_{2}r_{x}^{-\sqrt{1-D}}$$

where *C*_1_ and *C*_2_ are the constants.

According to the geometric equation between displacement and strain, the radial and hoop strains at any point in the cracked cylinder can be derived as [13,30]:

(51) $$\{\begin{array}{l} \varepsilon_{r}=C_{1}\sqrt{1-D}r_{x}^{\sqrt{1-D}-1}-C_{2}\sqrt{1-D}r_{x}^{-\sqrt{1-D}-1}\\ \varepsilon_{\theta }=C_{1}r_{x}^{\sqrt{1-D}-1}+C_{2}r_{x}^{-\sqrt{1-D}-1} \end{array}$$

Assuming that the thickness of each ring in the cracked cylinder is Δ*R*, the total thickness of the cracked cylinder is *N*Δ*R*. For the *N*th ring shown in Figure 11 adjacent to the uncracked concrete, its damage variable *D*^N^ is close to 0. Therefore, it can be assumed that the damage variable of the whole *N*th ring is 0, and the constants $C_{1}^{N}$ and $C_{2}^{N}$ in Equation (50) can be determined according to the condition that Equation (51) and Equation (39) are equal at the position of *r*_x_ = *R*_c_. When the constants in Equation (50) are determined, the radial and hoop strains at any point in the *N*th ring can be calculated according to Equation (51). Since the inner surface of the *N*th ring is the outer surface of the (*N*−1)th ring, for the (*N*−1)th ring, the constants $C_{1}^{N-1}$ and $C_{2}^{N-1}$ in Equation (50) can be derived according to the strain continuity condition of the two rings at the contact surface. The damage variable *D*^N−1^ of the (*N*−1)th ring can be determined by Equation (43), and the parameter *ε*_θ_ in Equation (43) takes the hoop strain on the inner surface of the *N*th ring. As a result, the radial and hoop strains at any point in the (*N*−1)th ring can also be calculated according to Equation (51). Similarly, the constants and strains from the (*N*−2)th ring to the 1th ring can be calculated. The above-detailed analysis process can be referred to in the literature [13]. According to the above analysis, the radial deformation of the concrete at the IBSC is equal to the radial displacement of the inner surface of the 1th ring, that is:

(52) $$\delta_{\mathrm{cc}}=u_{r}^{1}=C_{1}^{1}R^{\sqrt{1-D^{1}}}+C_{2}^{1}R^{-\sqrt{1-D^{1}}}$$

where *D*^1^ is the damage variable of 1th ring, which can be calculated according to Equation (43).

At the partial cracking stage of the concrete cover, the mass loss and corrosion depth of the steel bars can be derived according to Equations (30)–(37). When the cracks develop on the surface of the thick-walled cylinder, i.e., *R*_c_ = *R* + *C*, the critical corrosion depth of the steel bars can be determined as:

(53a) $$\delta_{\mathrm{steel}}^{\mathrm{surface}}=\frac{d-d_{\rho }}{2}$$

(53b) $$d_{\rho }=\sqrt{1-\eta_{s}^{\mathrm{surface}}}d$$

where $\eta_{s}^{\mathrm{surface}}$ is the critical mass loss of steel bars when the CCS cracks.

**Figure 12:**
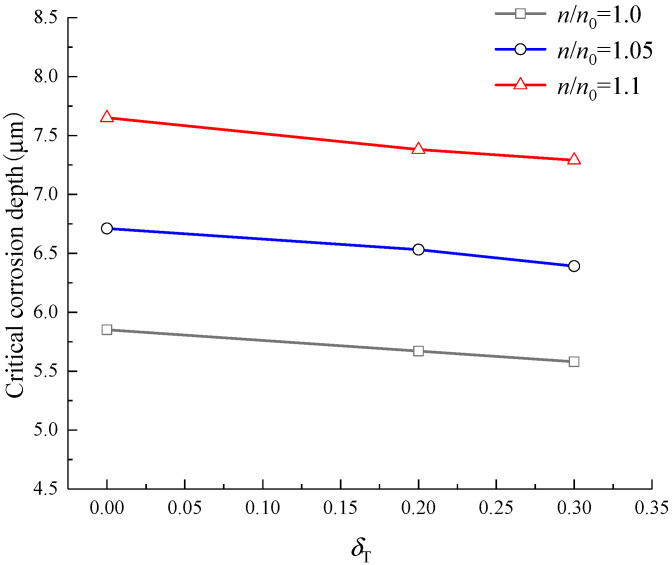
Critical corrosion depth of steel bars when the CCS cracks.

To analyse the influence of load on the critical corrosion depth of steel bars when the CCS cracks are induced by corrosion, the same example as Section 4 is used. The internal friction angle *φ* of concrete is 37°, the cohesion strength *c* = 5.01 MPa of concrete is calculated according to Equation (42), Δ*R* is 1 mm, and the values of *A*_t_ and *B*_t_ are 0.7 and 10^4^, respectively. Zhao et al. [13] found that when the cracks propagated to 0.8 times the thickness of the concrete cover, the corrosion expansion force at the IBSC reached the maximum, and at this time, even if the corrosion process of steel bars stopped, the cracks could spontaneously extend to the CCS. Therefore, this paper assumes that when the radius of the interface between the cracked cylinder and intact cylinder is *R*_c_ = *R* + 0.8*C*, the CCS cracks. Assuming that the hoop tensile stress at the position *r*_x_ = *R* of concrete cover caused by the radial pressure is 0, 0.2, and 0.3 times the tensile strength of concrete, that is, the tensile stress level *δ*_T_ = 0, 0.2, and 0.3, and meanwhile, the IBSC does not crack [54]. The range of *n*/*n*_0_ is about 1–1.2 [31], and 1.0, 1.05, and 1.1 are taken in this example. When the CCS cracks are induced by corrosion under load, the critical corrosion depth of steel bars is shown in Figure 12.

Figure 12 illustrates that the load affects the critical corrosion depth of the steel bars when the CCS cracks, but the effect is not significant. The larger the load, the smaller the critical corrosion depth of the steel bars. The larger the *n*/*n*_0_, the more rust filled into the pores of concrete around steel bars, resulting in a larger critical corrosion depth of the steel bars. For the three cases of *δ*_T_ = 0, 0.2, and 0.3, when the *n*/*n*_0_ increases from 1.0 to 1.1, the critical corrosion depth of steel bars increases by 30.8%, 30.2%, and 30.6%, respectively. For the three cases of *n*/*n*_0_ = 1.0, 1.05, and 1.1, when the tensile stress level *δ*_T_ increases from 0 to 0.3, the critical corrosion depth of the steel bars decreases by 4.6%, 4.8%, and 4.7%, respectively. Apparently, the value of *n*/*n*_0_, that is, the influence of the rust-filling layer on the critical corrosion depth of the steel bars is greater than that of the load. Compared with the analysis results of Section 4, it can be found that the influence of the load on the critical corrosion depth of the steel bars when the IBSC cracks is larger than that of when the CCS cracks.

## 6. Prediction Model of CLC Time of CCS under Load

According to the definition of the nominal rust volume expansion ratio *n*_0_ described in Section 3, when the CCS cracks, the thickness of the rust layer is:

(54) $$T_{\mathrm{CL}}^{\mathrm{surface}}=n_{0}\delta_{\mathrm{steel}}^{\mathrm{surface}}-\delta_{r}$$

Considering the thickness of the equivalent rust layer, when the CCS cracks, the total thickness of the rust layer is:

(55) $$d_{s}\left(t\right)=T_{\mathrm{CL}}^{\mathrm{surface}}+T_{\mathrm{CL},\mathrm{pore}}^{\mathrm{surface}}=T_{\mathrm{CL}}^{\mathrm{surface}}\left(1+\gamma k_{T}\right)=\left(n_{0}\delta_{\mathrm{steel}}^{\mathrm{surface}}-\delta_{r}\right)\frac{n}{n_{0}}=n\delta_{\mathrm{steel}}^{\mathrm{surface}}-\frac{n\delta_{r}}{n_{0}}$$

Liu and Weyers [16] found that after the steel bars in concrete were corroded, the total thickness of the rust layer when the corrosion time was *t* could be expressed as:

(56) $$\{\begin{array}{l} d_{s}\left(t\right)=\frac{W_{\mathrm{rust}}\left(t\right)}{2\pi R}\left(\frac{1}{\rho_{\mathrm{rust}}}-\frac{\alpha_{\mathrm{rust}}}{\rho_{\mathrm{st}}}\right)\\ W_{\mathrm{rust}}\left(t\right)=\sqrt{2\int_{0}^{t}\frac{0.21\pi Ri_{\mathrm{corr}}\left(t\right)}{\alpha_{\mathrm{rust}}}dt} \end{array}$$

where *α*_rust_ is the constant related to the type of corrosion products, which can be taken as 0.57 [16]; *ρ*_rust_ is the density of corrosion products, which can be taken as 3600 kg/m^3^ [16]; *ρ*_st_ denotes the density of the steel bars and is taken as 7850 kg/m^3^; *W*_rust_ (*t*) means the total amount of corrosion products at corrosion time *t*; and *i*_corr_ (*t*) represents the current density at corrosion time *t*.

When the current density of steel corrosion is a constant, the CLC time of CCS under load can be directly derived according to Equations (55) and (56):

(57) $$t_{\mathrm{cr}}^{\mathrm{surface}}=\frac{\alpha_{\mathrm{rust}}\pi R}{0.105i_{\mathrm{corr}}}{\left[\frac{n\delta_{\mathrm{steel}}^{\mathrm{surface}}-\frac{n\delta_{r}}{n_{0}}}{\frac{1}{\rho_{\mathrm{rust}}}-\frac{\alpha_{\mathrm{rust}}}{\rho_{\mathrm{st}}}}\right]}^{2}$$

When the current density of steel corrosion changes with time, the relationship between current density and time needs to be determined according to the measured values. Then, the relationship is substituted into Equation (56), followed by integration with respect to *t* from 0 to $t_{\mathrm{cr}}^{\mathrm{surface}}$. Finally, the CLC time of CCS under load can be determined according to Equations (55) and (56).

## 7. Model Validation

For the model established in Section 5, the rationality has been verified by Zhao et al. [30] when the influence of the load is not considered. This section focuses on verifying the rationality of the model considering the effect of load. Wang et al. [43] recorded the CLC time (i.e., initiation cracking time) of the CCS of recycled concrete beams under load as listed in Table 1, and in their test, the load applied to recycled concrete beams was 0.2, 0.4, and 0.6 times the flexural capacity, respectively. It can be found that the CLC time of the CCS of recycled concrete beams tends to decrease with the increase of the applied load. Due to the load borne by RC structures during the service stage not being larger than 0.6 times the flexural capacity [62], it can be concluded that the recycled concrete beams in the test of Wang et al. [43] are all in the service stage. It should be noted that Equation (57) is established based on the uniform corrosion of steel bars, whereas the corrosion of steel bars under load has obvious pitting corrosion characteristics. However, the electrochemical corrosion method is adopted in the corrosion test of Wang et al. [43]; hence, the corrosion of steel bars in recycled concrete beams under load is mainly uniform corrosion, which has been discussed in detail by Wang et al. [43]. Therefore, the experimental values of Wang et al. [43] can be adopted to verify the rationality of Equation (57). When the Equation (57) is adopted to calculate the CLC time of CCS under load, the values of the parameters in Equation (57) are as follows: *α*_rust_ = 0.57, *ρ*_rust_ = 3600 kg/m^3^, *ρ*_st_ = 7850 kg/m^3^, *R* = 6 mm, *n* = 2, *i*_corr_ = 0.01 mA/mm^2^, *n*/*n*_0_ = 1.1, *C* = 23 mm, *b* = 29 mm, *v*_c_ = 0.2, *φ* = 37°, *A*_t_ = 0.7, *B*_t_ = 10^4^, Δ*R* = 0.5 mm, and assuming *R*_c_ = 6 + 0.8 × 23 ≈ 25 mm when the CCS cracks.

The comparison of the calculated values from Equation (57) and the experimental values are listed in Table 1. It can be found that the calculated values are slightly larger than the experimental values, but the two are basically consistent. There are two main reasons for this phenomenon: on the one hand, in the test of Wang et al. [43], the steel bars embedded in recycled concrete beams are not completely rusted uniformly, and there are many local pits on the surface of steel bars, and it is well known that pitting corrosion of steel bars can shorten the CLC time of CCS; on the other hand, it is difficult to accurately determine the values of *n* and *n*/*n*_0_, and the analysis results of Section 4 and Section 5 show that the value of *n*/*n*_0_ has an effect on the critical corrosion depth of steel bars, i.e., the larger the *n*/*n*_0_, the larger the critical corrosion depth, which in turn affects the CLC time of CCS under load, hence the *n*/*n*_0_ = 1.1 is taken in Equation (57), which may be greater than the actual value of *n*/*n*_0_ for the recycled concrete beams in the test of Wang et al. [43].

## 8. Parametric Analysis

**Figure 13:**
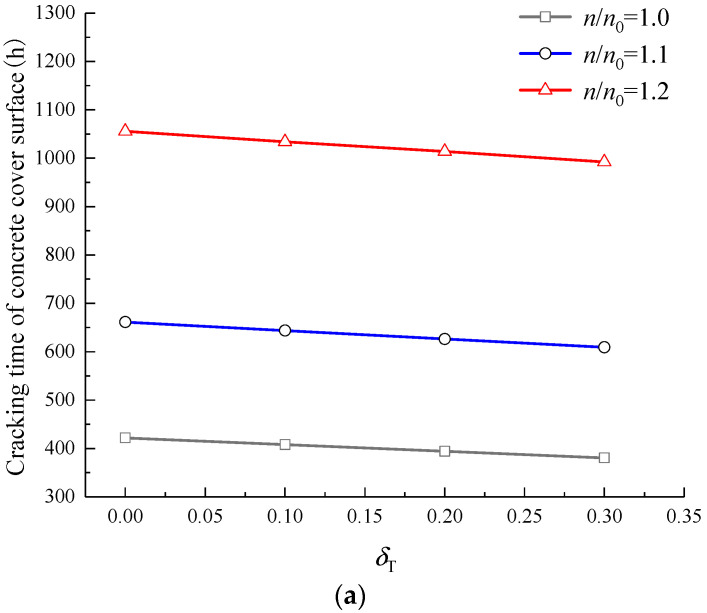

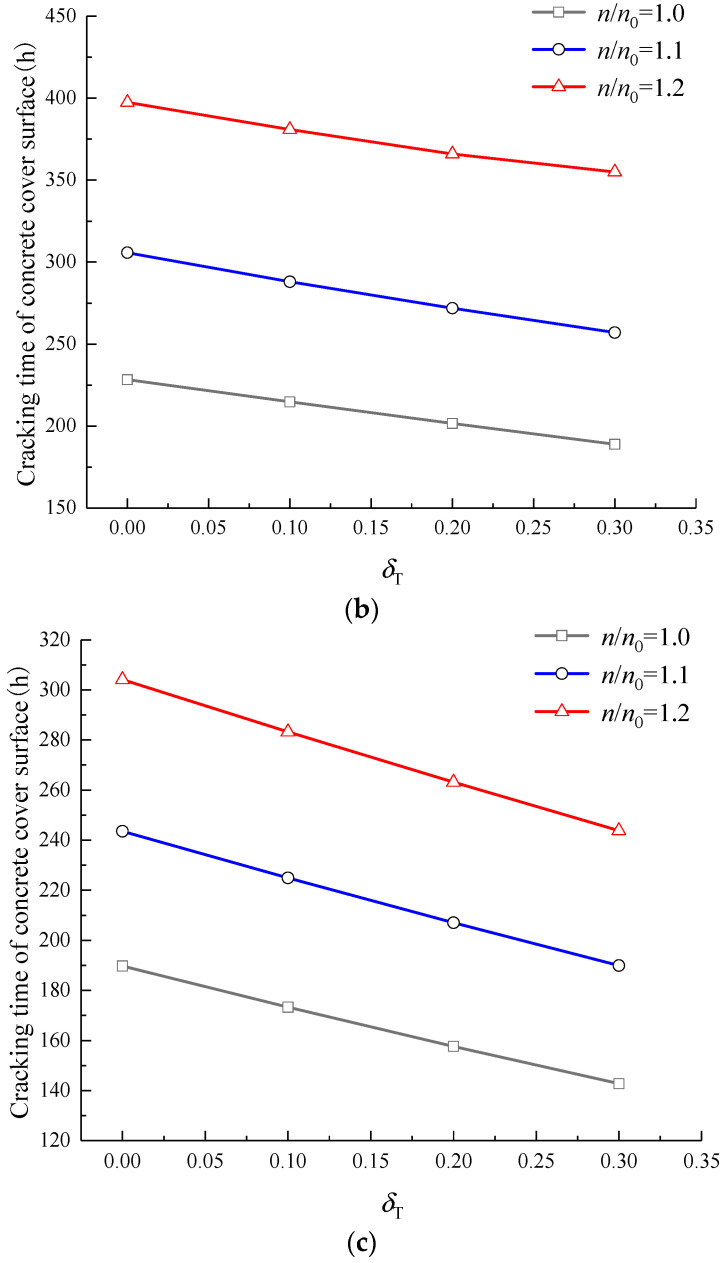
Effects of load, *n*, and *n*/*n*_0_ on the CLC time of CCS. (**a**) *n* = 2; (**b**) *n* = 3; (**c**) *n* = 4.

There are some factors affecting the CLC time (i.e., initiation cracking time) of CCS, such as concrete tensile strength, concrete cover thickness, reinforcement diameter, current density, rust property, etc. [14,49,60]. Based on the prediction model established in Section 6, this section analyses the influence of load, *n*, and, *n*/*n*_0_ on the CLC time of CCS. The RC beam as shown in Section 4 is adopted, the values of parameters are listed in Section 4 and Section 5, and the current density *i*_corr_ = 0.01 mA/mm^2^. Assuming that the hoop tensile stress at the position *r*_x_ = *R* of concrete cover caused by load is 0, 0.1, 0.2, and 0.3 times the tensile strength of concrete, the tensile stress level *δ*_T_ = 0, 0.1, 0.2, and 0.3. The range of the actual rust volume expansion ratio *n* is about 2–4 [13,14], and 2, 3, and 4 are adopted in this section. The range of *n*/*n*_0_ is about 1–1.2 [31], and 1.0, 1.1, and 1.2 are taken in this section. The influence of load, *n*, and *n*/*n*_0_ on the CLC time of CCS is shown in Figure 13.

Figure 13 shows that, with the increase of *n* or load, the CLC time of CCS decreases, while the CLC time increases obviously with the *n*/*n*_0_. For example, in the case of *n* = 3 and *n*/*n*_0_ = 1.2, when *δ*_T_ increases from 0 to 0.3, the CLC time decreases from 397.42 h to 355.04 h; while in the case of *n* = 4 and *δ*_T_ = 0.30, when *n*/*n*_0_ increases from 1.0 to 1.2, the CLC time increases from 142.78 h to 243.80 h. In order to further investigate the effect of load on CLC time, the influence of load on the CLC time of CCS can be calculated quantitatively according to Figure 13, as listed in Table 2. It can be found from Table 2 that the values of *n* and *n*/*n*_0_ affect the influence of load on the CLC time. When *n*/*n*_0_ remains unchanged, the greater the *n*, the more obvious the load can shorten the CLC time, that is, the percentage reduction in CLC time caused by load increases with *n*. For instance, in the case of *n*/*n*_0_ = 1.1, when *n* increases from 2 to 4, the percentage reduction in CLC time caused by the load is increased by 178.5%. However, when *n* remains unchanged, the percentage reduction in CLC time caused by the load decreases with the increase of *n*/*n*_0_. For instance, for the case of *n* = 3, when *n*/*n*_0_ increases from 1.0 to 1.2, the percentage reduction in CLC time caused by the load is decreased by 27.2%.

## 9. Conclusions

This paper studied the effect of load on the CLC time of CCS of RC structures, and the following conclusions can be drawn:

(1) The load has a significant effect on the critical corrosion depth of steel bars when the IBSC cracks induced by corrosion, and for the *n/n*_0_ = 1.0 and 1.2, when the tensile stress level increases from 0 to 0.3, the critical corrosion depth is reduced by 67.9% and 59.4%, respectively;
(2) The load does not have an obvious effect on the critical corrosion depth of the steel bars when the CCS cracks induced by corrosion, and for the *n*/*n*_0_ = 1.0, 1.05, and 1.1, when the tensile stress level increases from 0 to 0.3, the critical corrosion depth is decreased by 4.6%, 4.8%, and 4.7%, respectively;
(3) When the CCS cracks induced by corrosion under load, the influence of the rust-filling layer on the critical corrosion depth of steel bars is larger than that of load, and the effect of load on the critical corrosion depth of steel bars when the IBSC cracks is larger than that of when the CCS cracks;
(4) Considering the effects of load and the rust-filling layer, a prediction model of the CLC time of CCS is established, and the calculated values of the prediction model are in reasonable agreement with the experimental values;
(5) The values of *n* and *n*/*n*_0_ affect the influence of load on the CLC time. When *n*/*n*_0_ remains unchanged, the percentage reduction in CLC time caused by load increases with *n*. However, when the *n* remains unchanged, the percentage reduction in CLC time caused by load decreases with an increasing *n*/*n*_0_.

## Figures and Tables

**Table 1:** Comparison of calculated values and experimental values of CLC time of CCS under load.

Beam Label	RAC-0.2-50	RAC-0.2-100	RAC-0.4-50	RAC-0.4-100	RAC-0.6-50	RAC-0.6-100
Experimental values (h)	134–136	132–134	106–114	106–114	89–92	89–92
Calculated values (h)	144.87	135.94	120.77	115.01	102.52	97.13

**Table 2:** Effects of load on the CLC time of CCS.

*n* = 2	PRCT	*n* = 3	PRCT	*n* = 4	PRCT
*n*/*n*_0_ = 1.0	9.8%	*n*/*n*_0_ = 1.0	17.3%	*n*/*n*_0_ = 1.0	24.8%
*n*/*n*_0_ = 1.1	7.9%	*n*/*n*_0_ = 1.1	15.9%	*n*/*n*_0_ = 1.1	22.0%
*n*/*n*_0_ = 1.2	6.0%	*n*/*n*_0_ = 1.2	12.6%	*n*/*n*_0_ = 1.2	19.8%

Note: PRCT stands for percentage reduction in CLC time when *δ*_T_ increases from 0 to 0.3.

## Data Availability

The data presented in this study are available on request from the corresponding author.
